# Newly emerged enterovirus-A71 C4 sublineage may be more virulent than B5 in the 2015–2016 hand-foot-and-mouth disease outbreak in northern Vietnam

**DOI:** 10.1038/s41598-019-56703-5

**Published:** 2020-01-13

**Authors:** Son T. Chu, Kyousuke Kobayashi, Xiuqiong Bi, Azumi Ishizaki, Tu T. Tran, Thuy T. B. Phung, Chung T. T. Pham, Lam V. Nguyen, Tuan A. Ta, Dung T. K. Khu, Masanobu Agoh, An N. Pham, Satoshi Koike, Hiroshi Ichimura

**Affiliations:** 1 Department of Viral Infection and International Health, Graduate School of Medical Sciences, Kanazawa University, Kanazawa, 9208640 Japan; 2 Neurovirology Project, Tokyo Metropolitan Institute of Medical Science, Tokyo, 1568506 Japan; 3 Graduate School of Advanced Preventive Medical Sciences, Kanazawa University, Kanazawa, 9208640 Japan; 4 Outpatient Department, Vietnam National Hospital of Pediatrics, Hanoi, 10000 Vietnam; 5 Research Biomolecular for Infectious Disease Department, Vietnam National Hospital of Pediatrics, Hanoi, 10000 Vietnam; 6 Center for Pediatric Tropical Diseases, Vietnam National Hospital of Pediatrics, Hanoi, 10000 Vietnam; 7 Medical Intensive Care Unit, Vietnam National Hospital of Pediatrics, Hanoi, 10000 Vietnam; 8 Neonatal Intensive Care Unit, Vietnam National Hospital of Pediatrics, Hanoi, 10000 Vietnam; 9 Department of Virology, Institute of Tropical Medicine, Nagasaki University, Nagasaki, 8528523 Japan; 10 Department of Pediatrics, Hanoi Medical University, Hanoi, 10000 Vietnam

**Keywords:** Viral epidemiology, Viral pathogenesis, Paediatric research, Infection

## Abstract

Enterovirus-A71 (EV-A71) is a common cause of hand-foot-and-mouth disease (HFMD) and, rarely, causes severe neurological disease. This study aimed to elucidate the epidemiological and genetic characteristics and virulence of EV-A71 strains isolated from children diagnosed with HFMD. Rectal and throat swabs were collected from 488 children with HFMD in Hanoi, Vietnam, in 2015–2016. From 391 EV-positive patients, 15 EVs, including coxsackievirus A6 (CV-A6; 47.1%) and EV-A71 (32.5%, n = 127), were identified. Of the 127 EV-A71 strains, 117 (92.1%) were the B5 subgenotype and 10 (7.9%) were the C4 subgenotype. A whole-genome analysis of EV-A71 strains showed that seven of the eight C4a strains isolated in 2016 formed a new lineage, including two possible recombinants between EV-A71 C4 and CV-A8. The proportion of inpatients among C4-infected children was higher than among B5-infected children (80.0% vs. 27.4%; P = 0.002). The virulence of EV-A71 strains was examined in human scavenger receptor class B2 (hSCARB2)-transgenic mice, and EV-A71 C4 strains exhibited higher mortality than B5 strains (80.0% vs. 30.0%, P = 0.0001). Thus, a new EV-A71 C4a-lineage, including two possible recombinants between EV-A71 C4 and CV-A8, appeared in 2016 in Vietnam. The EV-A71 C4 subgenotype may be more virulent than the B5 subgenotype.

## Introduction

Hand-foot-and-mouth disease (HFMD) is an acute viral infectious disease that typically affects infants and children under 5 years of age. HFMD is usually mild and self-limited; however, severe HFMD is associated with neurological diseases, including rhombencephalitis, acute flaccid paralysis, and aseptic meningitis, which induce pulmonary oedema, cardiopulmonary dysfunction, and death^1^.

The first HFMD outbreak was reported in 1957 in Toronto, Canada^2^. Since that time, HFMD has become a continuing threat to global public health, particularly in the Asian-Pacific region^3^. Annually, millions of patients have contracted HFMD in Taiwan, China, Singapore, and the Republic of Korea^1,4^. In Vietnam, the first HFMD case was reported in 2003^5^, and the largest HFMD outbreak occurred in February 2011 to July 2012 with 174,677 cases and 200 deaths upon hospital admission^6^.

HFMD is caused by enteroviruses (EVs) of the family *Picornaviridae*. At least 22 EV serotypes have been identified in HFMD^7^; the three most frequently reported are EV-A71, coxsackievirus A16 (CV-A16), and CV-A6, all of which belong to human EV-A^5,6,8–10^. CV-A6, CV-A16, and other EVs are associated with mild symptoms that resolve within a few weeks. In contrast, EV-A71 is occasionally associated with severe outcomes^6,11^.

Based on the phylogenetic relationships of the VP1 gene, which encodes major viral capsid proteins, EV-A71 is classified into eight genotypes A–H^12–16^. Genotype A contains only the prototype strain (BrCr). Genotypes B and C have five subgenotypes each, B1–B5 and C1–C5, with the C4 subgenotype further divided into two lineages, C4a and C4b^17^. The genotype B prototype was found in the Netherlands as early as 1963, and the genotype C prototype was found in Japan as early as 1978^18,19^.

Four EV-A71 subgenotypes, C1, C4, C5, and B5, have been identified as causative agents of HFMD in Vietnam between 2003 and 2013^8^. Subgenotype C5 appeared in 2003 and was dominant until 2011, C1 circulated on a small scale in 2005, C4 circulated in 2004–2006 and from 2011 to the present, and B5 appeared in 2011 and became dominant in 2013^8,9,20^. In 2011–2012, the largest outbreak ever observed with a significant proportion of severe cases occurred simultaneously with the shift of the dominant EV-A71 subgenotype from C5 to C4^6^. In 2013, a small outbreak with a much lower proportion of severe cases occurred with another shift from C4 to B5^4,8^. In addition, many different subgenotypes of EV-A71 have circulated one after another, with C4 causing large outbreaks from 2008 to 2012 in China, B3 in 1997 in Malaysia, C2 in 1998 in Taiwan, and C4 in 2012 in Cambodia^21–24^. These observations suggest some difference in virulence among circulating genotypes or subgenotypes of EV-A71, which remains unclear.

Fujii *et al*. previously developed transgenic mice carrying the EV-A71 receptor, human scavenger receptor B class 2 (hSCARB2), which confers susceptibility to several causative agents of HFMD, including EV-A71, CV-A16, CV-A14, and CV-A7. This transgenic mouse (hSCARB2-tg) was suitable for assessing EV-A71 virulence without the bias of host and environmental factors^25^.

The present study aimed to elucidate the epidemiological and virological characteristics of EVs that cause HFMD in northern Vietnam. We tested the virulence of EV-A71 subgenotypes in the hSCARB2-tg mouse model.

## Results

### Enterovirus serotype prevalence

**Figure 1:**
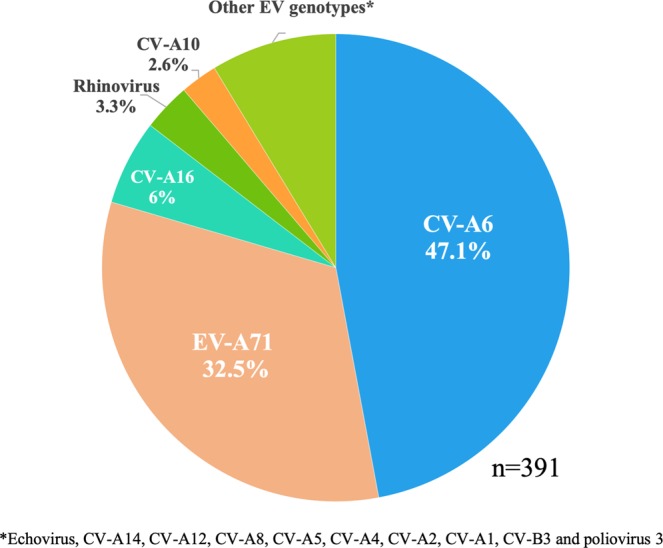
Detected enterovirus serotypes and their distribution among children with HFMD in 2015. Enterovirus serotypes were identified based on partial VP1 sequences in children with hand-foot-and-mouth disease (HFMD) in northern Vietnam in 2015–2016. CV: coxsackievirus, EV: enterovirus.

First, we analysed 488 clinical samples isolated from HFMD patients in northern Vietnam in 2015–2016 by a CODEHOP reverse transcription-semi-nested PCR (RT-snPCR) assay. We identified partial VP1 sequences in 442 (90.6%) of the 488 children with HFMD: in 383 children from their throat swabs and in 59 children from their rectal swabs collected from the 105 children whose throat swabs were negative for the RT-snPCR. The partial VP1 gene was successfully analysed in 391 of these 442 patients (88.5%; 92 inpatients and 299 outpatients). We identified 15 EV serotypes; CV-A6 (47.1%, 184/391) was most prevalent, followed by EV-A71 (32.5%, 127/391; Fig. 1).

### Phylogenetic analysis of EV-A71

**Figure 2:**
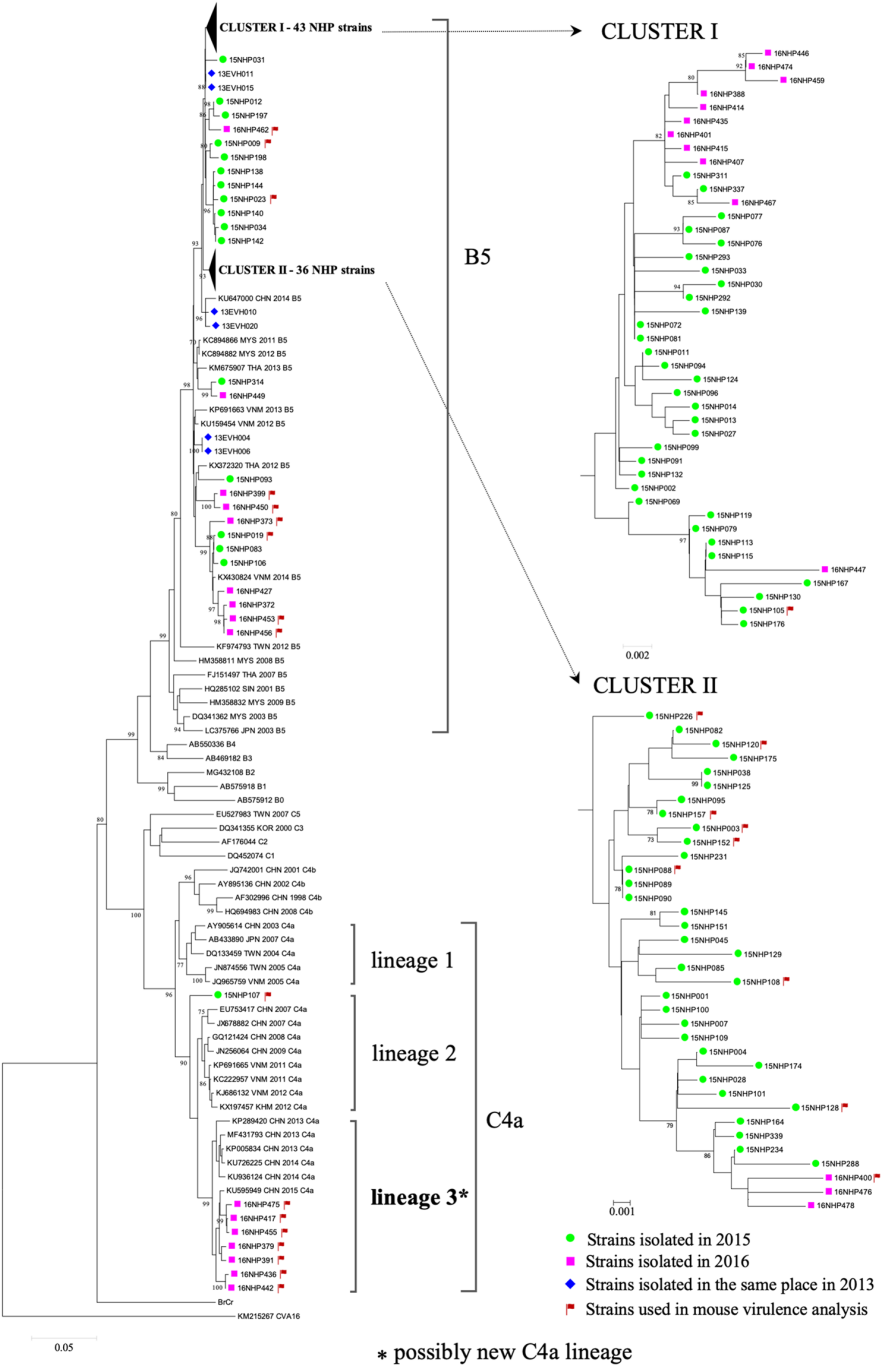
Phylogenetic tree of EV-A71 strains based on complete VP1 gene sequences. Blue diamond indicates the strains isolated in 2013, green circle indicates the strains isolated in 2015, and pink square indicates the strains isolated in 2016. Red flag indicates the strains used in mouse virulence analysis. *Indicates a possibly new C4a lineage. EV: enterovirus. VP1: viral protein 1.

From the 127 swabs (96 throat swabs and 31 rectal swabs) that were positive for EV-A71 in PCR assays, we isolated 117 EV-A71 strains in RDΔEXT1 + hSCARB2 cells: 86 strains (89.6%) from the throat swabs and 31 strains (100%) from the rectal swabs (P = 0.12). The entire VP1 gene was successfully sequenced in 112 isolates. A phylogenetic analysis based on the complete VP1 gene showed that 104 (92.9%) strains belonged to subgenotype B5 and 8 (7.1%) belonged to subgenotype C4 (Fig. 2). Seven of the eight C4 strains were isolated in 2016. These formed a putative new C4a lineage that clustered with the C4 strains isolated in China in 2013–2015. The mean p-distances between the putative new C4a lineage and C4a lineages 1 and 2 were 3.5% and 4.9%, respectively. The remaining C4 strain was isolated in 2015 (15NHP107) and identified as an outlier from C4a lineages 1 and 2 and from the putative new lineage.

### Recombination analysis of EV-A71 strains

**Figure 3:**
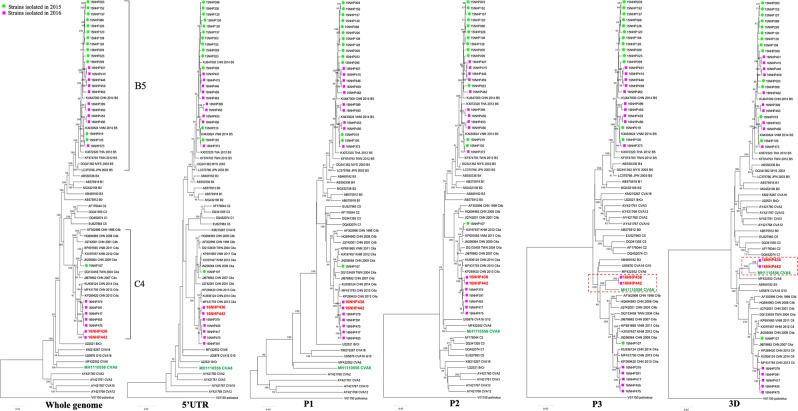
Phylogenetic trees of 31 EV-A71 strains based on the full genome sequences. The trees were constructed from aligning the whole genome and the 5′ UTR, P1, P2, P3, and 3D by the neighbour-joining method. Bootstrap values were calculated from 1000 replicates. Green circle indicates the strains isolated in 2015, and pink square indicates the strains isolated in 2016. EV: enterovirus, UTR: untranslated region.

We conducted whole-genome sequencing to clarify the evolution of the eight C4a strains and 23 representative B5 strains classified based on the complete VP1 gene sequences. Phylogenetic trees were constructed based on the whole genome and the 5′UTR, P1, P2, P3, and 3D regions of the 31 strains. The EV reference sequences were retrieved from Genbank. In the phylogenetic trees, two C4a strains (16NHP436, 16NHP442) formed a cluster with CV-A8 in the P3 and 3D regions (Fig. 3). We found no phylogenetic incongruence among the 23 B5 strains for all genomic regions.

**Figure 4:**
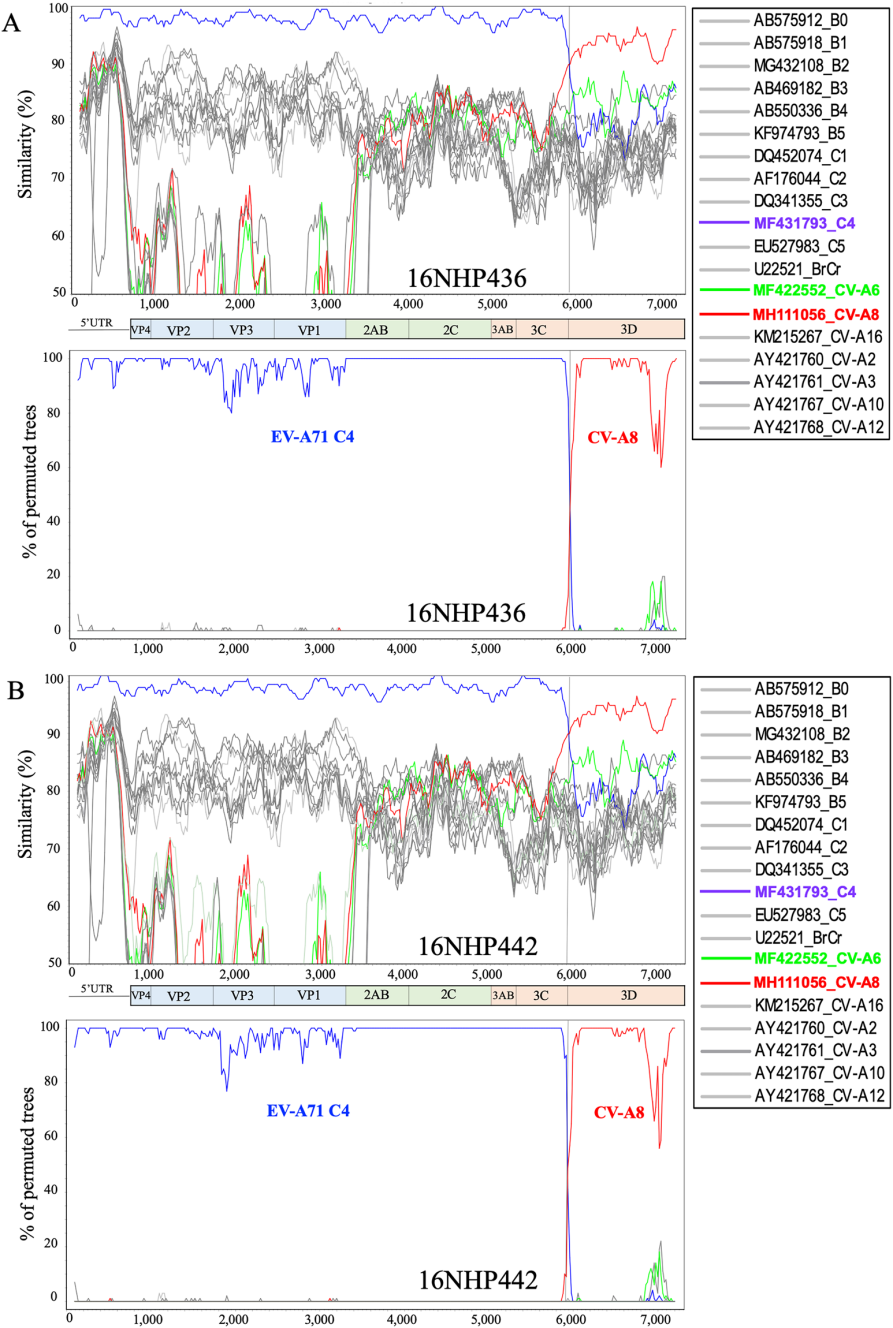
Recombination analysis of EV-A71 strains 16NHP436 and 16NHP442 isolated in northern Vietnam in 2016. (**A**) EV-A71 strain 16NHP436 was the query sequence. (**B**) EV-A71 strain 16NHP442 was the query sequence. Similarity plots (upper panels) and bootscan analyses (lower panels) were created using SimPlot version 3.5.1 (Kimura distance model; window size 200, step size 20, and 100 bootstrap replicates). The blue lines denote EV-A71 subgenotype C4, the red lines denote the CV-A8 genotype, and the green lines denote the CV-A6 genotype. Dashed vertical lines indicate potential recombination breakpoints. EV: enterovirus, CV: coxsackievirus.

The 16NHP436 and 16NHP442 strains were further investigated with similarity plot and bootscan analyses (Fig. 4). Sequence analysis of these two strains showed evidence of a genomic recombinant structure, as their nucleotide sequences in the region between nucleotides 1–5930 including the 5′UTR, P1, P2, 3A, 3B, and 3C sequences were similar to other contemporary EV-A71 C4 strains, as expected, but those from nucleotide 5,930 (3D region) were genetically closer to a CV-A8 strain from Australia (2017). Thus, recombination could have occurred between EV-A71 and a CV-A8 strain or between EV-A71 and any other common ancestor strain of these EV-A71 strains and the CV-A8 strain from Australia, including a strain from a different enterovirus A serotype.

### Evaluation of the neurovirulence of circulating viruses using human clinical data

EV-A71 is known to be more neurovirulent or neuroinvasive than other species A EVs^6,11^, and patients with grade 2 and above have neurological signs and/or symptoms. Therefore, the proportion of inpatients may be a means to evaluating the neuroinvasiveness of the circulating viruses using human clinical data. The proportion of inpatients was significantly higher among EV-A71-infected children with HFMD than CV-A6-infected children with HFMD (31.5% vs. 15.2%, P = 0.001; Table 1). This result suggests that EV-A71 may be more neurovirulent than CV-A6.

**Table 1:** Relationship between EV-A71 and CV-A6 genotypes and disease severity.

EV genotype	Outpatients	Inpatients			P value
	Grade 1	Grade 2	Grade 3	Grade 4	
EV-A71 (n = 127)	87 (68.5%)	38 (29.9%)	0	2 (1.6%)	0.001
CV-A6 (n = 184)	156 (84.8%)	28 (15.2%)	0	0	

P-value was calculated by the chi-squared test.

Next, we analysed whether a difference in neurovirulence exists among the observed subgenotypes of EV-A71. We identified EV-A71 subgenotypes based on the partial and/or complete VP1 gene sequence; of 127 EV-A71 strains detected, 117 were subgenotype B5 (85 [72.6%] outpatients and 32 [27.4%] inpatients) and 10 were subgenotype C4a (2 [20.0%] outpatients and 8 [80.0%] inpatients; Table 2). The proportion of inpatients among C4-infected patients was significantly higher than among B5-infected patients (P = 0.002). Although the number of patients is relatively small, the result suggests the possibility that EV-A71 subgenotype C4 may be more neurovirulent than EV-A71 subgenotype B5.

**Table 2:** Relationship between EV71 subgenotypes and disease severity.

Subgenotype	Outpatients	Inpatients			P value
	Grade 1	Grade 2	Grade 3	Grade 4	
B5 (n = 117)	85 (72.6%)	30 (25.7%)	0	2 (1.7%)	0.002
C4 (n = 10)	2 (20.0%)	8 (80.0%)	0	0	

P-value was calculated by the chi-squared test.

### Virulence of EV-A71 C4 and B5 subgenotypes in hSCARB2-tg mice

**Figure 5:**
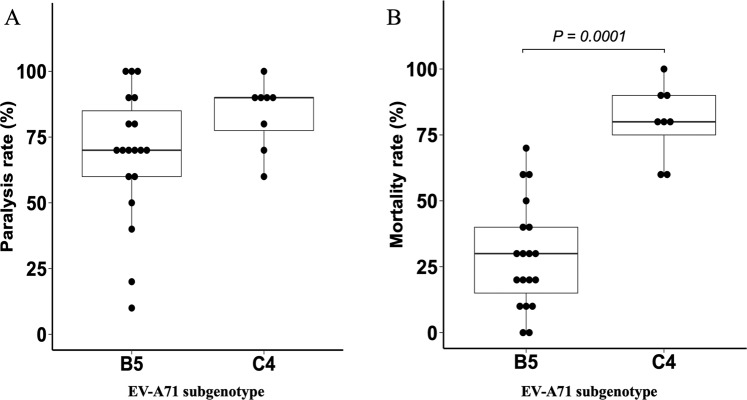
Paralysis and mortality rates of hSCARB2-tg mice infected with EV-A71 B5 and C4. hSCARB2-tg mice were inoculated with EV-A71 B5 (19 strains) and C4 (8 strains) intraperitoneally at 5 × 10^5^ TCID_50_. (**A**) Paralysis rates 14 days post-infection. (**B**) Mortality rates 14 days post-infection. P-values were calculated by the Mann-Whitney U test. EV: enterovirus, hSCARB2-tg mice: mice that carry the human scavenger receptor class B2 transgene.

To experimentally assess the virulence of the virus strains, 19 EV-A71 B5 strains and 8 C4 strains were inoculated into hSCARB2-tg mice (Fig. 5). The amino acid residue of VP1–145 of all clinical isolates used in this study was glutamic acid. We found that mutations of this residue to glycine or glutamine are sometimes observed after passages in cultured cells. We also found that these mutations were associated with attenuation of the virus in hSCARB2-tg mice even though the contamination rate of these mutants was low (Kobayashi *et al*. submitted for publication). We therefore used the virus stocks that contained the mutants less than 0.1% estimated by the next generation sequencing (NGS) analysis to avoid the bias caused by the contamination (Supplementary Table S1). The virus strains used in this study were highly homogenous with regard to the amino acid residue VP1–145.

We assessed the virulence of the viruses by the clinical signs and survival of the hSCARB2-tg mice. The mice inoculated with EV-A71 began to show neurological signs such as limb weakness associated with wobbling and flaccid paralysis 3–7 days after infection, and were recognized to be paralyzed. The paralysis became severe in some mice, which died or were sacrificed due to the humane endpoint (complete paralysis in more than two limbs or moribund). In other mice, the paralysis was not evident, and they recovered from the weakened limbs and body weight loss. The body weight change and clinical signs of individual mouse are shown in Supplementary Fig. S1. Although the clinical signs caused by the viruses of two different subgenotypes were not distinguishable, mortality rates were significantly higher in hSCARB2-tg mice infected with C4 strains (median [range]: 80.0% [60.0–100.0%]) than those infected with B5 strains (30.0% [0–70.0%], P = 0.0001). Paralysis rates were also slightly higher in hSCARB2-tg mice infected with C4 strains than those infected with B5 strains, though the difference was not significant (90.0% [60.0–100.0%] vs. 70.0% [10.0–100.0%], P = 0.124)(Fig. 5). No mock-infected hSCARB2-tg mice had paralysis or death for 14 days after inoculation (data not shown). We also analysed whether the virulence of the B5 strains differs depending on the clinical grades of the patients. However, we found no significant difference in mortality rates and paralysis rates between hSCARB2-tg mice infected with B5 strains isolated from HFMD children with clinical grade 1 (outpatients) or 2 (inpatients) (Supplementary Fig. S2).

16NHP436 and 16NHP442 strains, which have a recombinant genome between EV-A71 C4 and CV-A8, exhibited similar virulence to other C4 strains in hSCARB2-tg mice (60% and 90% mortality rate, and 70% and 90% paralysis rate, respectively). Therefore, a distinct effect of the recombination on EV-A71 neurovirulence was not observed, at least in these two strains.

## Discussion

This study provided a comprehensive analysis of the epidemiological and virological characteristics of the EVs, particularly EV-A71, that caused HFMD in northern Vietnam in 2015–2016. This was the first report to use hSCARB2-tg mice to compare the virulence of clinical isolates belonging to two different EV-A71 subgenotypes, C4 and B5, that circulated during the same outbreak.

Here, we demonstrated the diversity of agents causing HFMD in Vietnam, identifying 15 EV serotypes. CV-A6 was detected most frequently, followed by EV-A71 and CV-A16. Our results are consistent with previous studies, which reported that CV-A6 displaced EV-A71 and CV-A16 and became the predominant EV serotype in China, Singapore, Thailand, and several European countries soon after the appearance of CV-A6 in Finland in 2008^7,26–30^.

Our phylogenetic analyses based on the complete VP1 gene showed that EV-A71 B5 mainly circulated in 2015–2016. In 2016, seven C4a strains in northern Vietnam formed a new cluster with the Chinese strains isolated in 2013–2015. This newly emerged C4a cluster was designated C4a-lineage 3, as the EV-A71 C4 subgenotype is classified as C4a and C4b, and C4a was further classified into two lineages, lineage 1 and lineage 2. EV-A71 is reportedly associated with severe neurological complications^11^. In this study, inpatients with clinical grade 2 and above were considered severe cases because they had neurological complications, shock symptoms, and/or other symptoms that required hospitalization. Consistent with the previous study^11^, the proportion of inpatients was significantly higher among HFMD patients infected with EV-A71 than those with other EVs. We also found a significantly higher proportion of inpatients among the patients infected with EV-A71 C4 than those infected with B5 (80.0% vs. 27.4%; P = 0.002), suggesting that the EV-A71 C4 subgenotype may be more virulent than B5, at least in Vietnamese children. This tendency was determined from the analysis using a small number of patients, especially the number of C4-infected patients, which was as few as eight. However, it was impossible to obtain more patients because the number of patients in the same area in the same year was limited.

Therefore, we employed another strategy to evaluate the virulence of the circulating viruses. The hSCARB2-tg mouse model is a useful tool for analysing the virulence of EV-A71 because we can precisely control the experimental conditions using the mice with homogeneous genetic backgrounds^25^. We found significantly higher mortality among transgenic mice infected with the C4 strains than those infected with the B5 strains. We selected 10, 8, and 1 strains from grade 1, grade 2, and grade 4 B5-infected patients, respectively. However, because of the limited number of samples, we used all eight isolated strains from the C4-infected patients; one and seven were from patients with grade 1 and grade 2, respectively. This asymmetrical selection might have created bias in the analysis. However, we observed no significant difference in mortality among transgenic mice infected with the B5 strains according to clinical grade (Supplementary Fig. S2). This result suggests that the EV-A71 C4 subgenotype may be more virulent than B5, which is consistent with our clinical findings.

In Vietnam, a shift in the dominant strain from EV-A71 C5 to C4 occurred in 2011. In 2011–2012, the largest HFMD outbreak with severe cases was observed^6^. In 2013, the reported number of cases and proportion of patients with complications declined, with another shift in dominance from C4 to B5^4,8^. In this study, most C4a strains were detected in severe cases. Moreover, in China, a C4 infection-related HFMD outbreak with more than seven million patients, including 2,457 fatal cases, occurred in 2008–2012^23^. Thus, it is possible that a shift of the dominant EV-A71 subgenotype from B5 to the new C4a lineage may lead to another outbreak with a worse outcome. Continuous monitoring of the causative agents of HFMD, particularly the EV-A71 strains, is necessary.

EVs expand their genetic diversity through both the accumulation of spontaneous mutations during replication and intratypic (e.g., between EV-A71 subgenotypes B and C) and intertypic (e.g., between EV-A71 and CV-A8 or CV-A16) recombination events^31–34^. Preferential recombination sites are in the 5′UTR and non-structural protein coding regions, such as P2 and P3^33,35^. In this study, we found two possible intertypic recombinant strains in which EV-A71 C4 was recombined with CV-A8 in the 3D region. Both of these strains were isolated from severe cases with grade 2. Recombination events might enhance their host range and virulence^24,33,35^, but the recombinant EV-A71 strains with the CV-A8 sequence had similar virulence as the other C4 strains in hSCARB2-tg mice. Further study is needed to monitor the new recombinant strains and assess their virulence.

HFMD has become a critical health problem in children worldwide, particularly in the Asian-Pacific region. Severe cases are mainly caused by EV-A71^11^, and there is no specific treatment. Thus, the development of vaccines against EV-A71 is the most effective approach for preventing children from contracting EV-A71 infections with severe symptoms. Several candidate EV-A71 vaccines have been developed using live attenuated strains, inactivated whole-virus particles, virus-like particles, recombinant proteins, and peptides^36^. In China, three types of inactivated vaccines using whole EV-A71 C4a particles have been commercially available since 2015^37^. However, it might be possible to identify more suitable candidate vaccine strains among the newly emerged EV-A71 C4a strains, especially for developing live attenuated vaccines.

This study had several limitations. First, small numbers of patients had grades 3 and 4 disease; the majority of severe cases (inpatients) were grade 2. These small numbers could restrict the significance of our clinical assessments of the virulence of EV-A71 subgenotypes. Second, only eight EV-A71 C4 strains were successfully isolated and tested for virulence in the hSCARB2-tg mouse model. Thus, further study is necessary to isolate more EV-A71 C4 strains and assess their virulence in hSCARB2-tg mice compared to B5 strains. Third, we did not investigate host factors that might be associated with disease severity. This latter investigation is planned for a future study.

In conclusion, our findings improved our understanding of the epidemiological and virological characteristics of EVs, particularly EV-A71, which caused the HFMD outbreak in 2015–2016 in northern Vietnam. A new EV-A71 C4a-lineage (lineage 3) and two possible recombinant viruses between EV-A71 C4a and CV-A8 appeared in 2016. The newly emerged C4a-lineage may be more virulent than the B5 strain that circulated in the 2015–2016 HFMD outbreak.

## Methods

### Subjects and disease grade definition

From 1 January 2015 to 31 December 2016, all children diagnosed with HFMD at Vietnam National Hospital of Pediatrics, Hanoi (NHP), were invited to participate in this study. Of 1,047 children diagnosed with HFMD, 519 children and their parents or legal guardian(s) gave their informed consent to take part in this study. Thirty-one children with chronic diseases, congenital, and pre-existing conditions were excluded from the study. Thus, 488 children diagnosed with HFMD (male/female: 331/157, median age: 17 months [range: 2–88]) were included in this study.

According to the 2012 Vietnamese guidelines for HFMD diagnosis and management, clinical severity is classified into four grades^38^. Grade 1 is the presence of herpangina and/or skin rash only; grade 2 is the presence of myoclonic jerk; grade 3 is the presence of autonomic dysfunction, with intractable fever and shock symptoms in the early stages; and grade 4 is the presence of cardiopulmonary collapse. Patients with grade 1 were followed at home; those with grades 2 and above received hospitalized care.

### Collection, detection, and genotyping of enteroviruses

Throat and rectal swabs were collected from patients diagnosed with HFMD in 2015 and 2016 using cotton tips soaked in phosphate buffered saline (PBS). The swabs were rinsed in 1 mL PBS and stored at −80 °C until use.

Samples were genotyped by analysing the VP1 gene sequence. Briefly, samples were centrifuged at 14,000 rpm for 15 min. RNA was extracted from 140 μL of rectal swab supernatants with the QIAamp viral RNA mini kit (QIAGEN GmbH, Germany) and from 100 μL of throat swab supernatants with the SMITEST EX-R&D kit (Medical & Biological Laboratories Co. Ltd, Nagoya, Japan), according to the manufacturer’s instructions. The partial VP1 gene of EVs was amplified in a RT-snPCR assay with Consensus-Degenerate Hybrid Oligonucleotide Primers (CODEHOP)^39^. The PCR products were sequenced using the BigDye Terminator v.1.1 Ready Reaction Cycle Sequencing Kit (Thermo Fisher Scientific, TX, USA). For virus classification and genotyping, BLAST was used to search the reference database of the National Center for Biotechnology Information (NCBI). Rectal swab samples were used for the analyses only when the partial VP1 gene of EVs was not detected in the throat swabs.

### Virus isolation

The amino acid residues at VP1–145 of all EV-A71 strains detected in the clinical samples were glutamic acid (data not shown). This amino acid residue quickly changed into glycine or glutamine, and the mutated viruses were attenuated when the viruses were propagated in RD-A cells, which express heparan sulphate at the cell surface. To minimize this mutation and attenuation, we used RDΔEXT1 + hSCARB2 cells, which are rhabdomyosarcoma cells lacking heparan sulphate expression by knocking out the EXT1 gene and introducing human SCARB2 cDNA by retrovirus-mediated gene transfer^40^, for virus isolation and preparation. RDΔEXT1 + hSCARB2 cells were maintained in Dulbecco’s modified Eagle medium (DMEM; Wako, Osaka, Japan) supplemented with 5% foetal bovine serum (FBS; JRH Biosciences, KS, USA) containing 1 μg/mL puromycin. RDΔEXT1 + hSCARB2 cells were inoculated with the filtered supernatants of swabs positive for EV-A71 RNA, incubated for 1 h at 37 °C, and cultured in DMEM supplemented with 2% FBS without puromycin. The cells and culture medium were harvested when cytopathic effects appeared. The virus was isolated by subjecting cells to three freeze-thaw cycles, then centrifugation at 14,000 rpm for 15 min. The supernatants were collected, aliquoted, and stored at −80 °C until use.

### Complete VP1 gene sequencing

Viral RNA was extracted from the isolated viruses using a QIAamp viral RNA mini kit (Qiagen GmbH, Germany). RT-PCR was performed in an Access RT-PCR System (Promega Co., Madison, WI, USA). Reactions were carried out using 2 μL of each viral RNA and 50 pmol of the primer pair: 2349 F (5′-GCYTAYATAATAGCAYTGGCGGCAGC-3′) and 3393 R (5′-CACCCGTTGAADTCYCACCARTTGGCG-3′). Each reaction was performed using the following protocol: 48 °C for 45 min; 94 °C for 2 min; 35 cycles of 94 °C for 10 s, 50 °C for 10 s, and 65 °C for 1 min; and then 65 °C for 5 min. Amplicons were directly sequenced using primers 2757 F (5′-GCHAAYTGGGAYATAGACATAAC-3′) and 3046 R (5′-CCAAAATRCTGCCYATRGG-3′). Sequences were analysed using an ABI 3730 (Applied Biosystems).

### Phylogenetic analysis

A phylogenetic tree was constructed using the neighbour-joining method in the Molecular Evolutionary Genetics Analysis program, version 6.0 (MEGA 6.0). The reconstructed evolution tree was validated by bootstrapping with 1000 replicates. EV-A71 subgenotypes A, B, and C and other EV sequences retrieved from Genbank were used as references.

### Whole-genome sequencing analysis

NGS libraries were prepared from viral RNAs using the NEBNext RNA Library Prep Kit for Illumina (NEB) and the NEBNext Multiplex Oligos for Illumina Dual Index Primer Set 1 (NEB) according to the manufacturer’s instructions, with an average fragment length of 300 bp. The libraries were sequenced on MiSeq (Illumina) using a 150 bp paired-end kit.

Adaptor and low-quality sequences were trimmed from raw sequence reads using PRINSEQ software version 0.20.4 (http://prinseq.sourceforge.net/index.html). To remove reads derived from host cells, the remaining reads were mapped to the hg38 human reference genome using Bowtie 2 software version 2.3.4.1 with default settings^41^. The consensus viral genomic sequence was assembled from the hg38 unmapped reads using a *de novo* assembler IVA software version 1.0.8 with default settings^42^.

To detect the abundance of the VP1–145 mutation, the hg38 unmapped reads were mapped to the assembled consensus sequence using Smalt software version 0.7.6 (https://www.sanger.ac.uk/science/tools/smalt-0) and converted to BAM files using Samtools software version 1.9–4-gaelf9d8. Generated BAM files were used for variant calling using Lofreq software version 2.1.2^43^. Single nucleotide variants (SNVs) were annotated using SnpEff software version 4.3t^44^. To ensure the accuracy of SNV data, SNVs that met the following criteria were discarded: the abundance ratio was less than 10^−3^ and the coverage at the position was less than log (1 – *p*)/log (1 – *f*) – 1, where the mutation frequency *f* was detected with a probability *p* or better^45^. In this study, *p* was set to 0.95. Number of reads mapped to the consensus viral sequence and average depth are shown in Supplementary Table S1.

Phylogenetic trees were constructed based on the full genome and on each of the 5′UTR, P1, P2, P3, and 3D regions using the neighbour-joining method. Similarity plot and bootscan analyses were conducted to detect possible recombination events. A clear cross-over of two query-reference profiles with sharp slopes was considered a swap of the best-fit reference and the presence of a nearby recombination breaking point.

### Virus challenge in hSCARB2-tg mice

We randomly selected 25 EV-A71 strains isolated from the B5-infected patients with HFMD clinical grade 1 and grade 2, including both strains from the patients with grade 4. However, we excluded some virus strains that did not grow to high titre suitable for inoculation into the hSCARB2-tg mice. We also excluded four virus strains containing the mutation at VP1–145 more than 0.1% estimated by the NGS analysis (Supplementary Table S1) because this mutation was selected during propagation of EV-A71 in cultured cells and severely decreased the virulence of EV-A71 (Kobayashi *et al*., submitted for publication). We finally selected 19 EV-A71 B5 strains: 10 strains isolated from the patients with grade 1, 8 from the patients with grade 2, and 1 from the patient with grade 4. Similarly, we selected eight C4 strains – one and seven strains from the patients with grade 1 and grade 2, respectively. These 19 B5 strains and the 8 C4 strains were inoculated into hSCARB2-tg mice. We used 6- or 7-week-old hSCARB2-tg mice^23^ for infection experiments (n = 10 per group). After anaesthetization by inhalation of isoflurane, a total of 500 µL of virus solution (5 × 10^5^ TCID_50_) and PBS as a mock control were inoculated intraperitoneally. Infected and mock-infected mice were monitored daily for paralysis (limb weakness with wobbling or complete flaccid paralysis) and death for 14 days. Mice with flaccid paralysis in more than two limbs, moribund, and/or more than 30% body weight loss were sacrificed by overdose of isoflurane or cervical dislocation.

### Ethics statements

This study protocol was reviewed and approved by the Biomedical Research Ethics Committee of the National Hospital of Pediatrics and Research Institute for Child Health, Vietnam (approval number [no.] 14–012), and conducted in compliance with the Vietnamese National Ethical Guidelines for Biomedical Research Involving Human Subjects, 2013, setup by the Ministry of Health, Vietnam. Written informed consent for the use of their clinical samples was obtained from the parents or legal guardian(s) of the sick children whose samples were analyzed. This study protocol was also approved by the Ethics Review Committees of the Kanazawa University, Japan (approval no. 1611–1) and of the Tokyo Metropolitan Institute of Medical Science, Japan (approval no. 14–34), based on the Ethical Guidelines for Medical and Health Research Involving Human Subjects, setup by the Ministry of Education, Culture, Sports, Science and Technology and the Ministry of Health, Labour and Welfare, Japan in 2014 (revised in 2017).

Animal experiments were carried out in accordance with the Guidelines for the Care and Use of Animals (Tokyo Metropolitan Institute of Medical Science, 2011), which follow the Fundamental Guidelines for Proper Conduct of Animal Experiments and Related Activities in Academic Research Institutions, setup by the Ministry of Education, Culture, Sports, Science and Technology, Japan in 2006. The protocol of animal experiments was approved by the Animal Use and Care Committee of the Tokyo Metropolitan Institute of Medical Science, Japan (approval no. 18047).

### Statistical analysis

Statistical analyses were performed using SPSS version 22. The chi-squared test was used to analyse differences in the proportions of categorical variables. The Mann-Whitney U test was used to test differences in the medians of continuous variables. P-values < 0.05 were considered significant.

### Nucleotide sequence accession number

The complete VP1 sequences of the 112 EV-A71 strains described here are registered in Genbank under accession numbers MH557097–MH557208.

### Disclaimer

The findings and conclusions of this report are those of the authors and do not necessarily represent Kanazawa University, the Tokyo Metropolitan Institute of Medical Science, Vietnam National Hospital of Pediatrics, or Hanoi Medical University.

## Supplementary information

Supplementary figures and table.
