# Supplementary material for: Newly emerged enterovirus-A71 C4 sublineage may be more virulent than B5 in the 2015–2016 hand-foot-and-mouth disease outbreak in northern Vietnam
Source: Sci Rep. 2020 Jan 13;10:159. doi: 10.1038/s41598-019-56703-5 (PMC6957505; doi:10.1038/s41598-019-56703-5)
Supplement: Supplementary file 1 — Supplementary figures and table. [file 41598_2019_56703_MOESM1_ESM.pdf]

Newly emerged enterovirus-A71 C4 sublineage may be more virulent than B5 in the 2015–2016 hand-foot-and-mouth disease outbreak in northern Vietnam

Son T Chu[1+], Kyousuke Kobayashi[2+], Xiuqiong Bi[1], Azumi Ishizaki[1,3], Tu T Tran[4], Thuy T B Phung[5], Chung T. T. Pham[5], Lam V Nguyen[6], Tuan A Ta[7], Dung T K Khu[8], Masanobu Ago[9], An N Pham[6,10], Satoshi Koike[2], and Hiroshi Ichimura[1,3*]

[1]Department of Viral Infection and International Health, Graduate School of Medical Sciences; Kanazawa University, Kanazawa, 9208640, Japan; [2]Neurovirology Project, Tokyo Metropolitan Institute of Medical Science, Tokyo, 1568506, Japan; [3]Graduate School of Advanced Preventive Medical Sciences; Kanazawa University, Kanazawa, 9208640, Japan; [4]Outpatient Department, Vietnam National Hospital of Pediatrics, Hanoi, 10000, Vietnam; [5]Research Biomolecular for Infectious Disease Department, Vietnam National Hospital of Pediatrics, Hanoi, 10000, Vietnam; [6]Center for Pediatric Tropical Diseases, Vietnam National Hospital of Pediatrics, Hanoi, 10000, Vietnam; [7]Medical Intensive Care Unit, Vietnam National Hospital of Pediatrics, Hanoi, 10000, Vietnam; [8]Neonatal Intensive Care Unit, Vietnam National Hospital of Pediatrics, Hanoi, 10000, Vietnam; [9]Department of Virology, Institute of Tropical Medicine, Nagasaki University, Nagasaki, 8528523, Japan; [10]Department of Pediatrics, Hanoi Medical University, Hanoi, 10000, Vietnam

Supplementary Figure S1. Body weight change of hSCARB2-tg mice was daily followed up for 14 days post infection with EV-A71. hSCARB2-tg mice were inoculated with EV-A71 B5 (19 strains) and C4 (eight strains) intraperitoneally at 5 x 10^5 $TCID_{50}$. The viruses were islolated from the patients with diffrent clinical grade. Of them, 10, 8 and 1 strains were from grade 1, grade 2 and grade 4 of B5-infected patients, respectively. Among eight strains of C4, one was from the patient with grade 1 and seven from those with grade 2. **x** denotes the death mice, **x** denotes the sacrificed mice, denotes the mice with paralysis, denotes the mice with no clinical symptom. EV: enterovirus, hSCARB2-tg mice: mice that carry the human scavenger receptor class B2 transgene.

Supplementary Figure S2. **Paralysis and mortality rates of hSCARB2-tg mice infected with EV-A71 B5 isolated from patients with different clinical grades.** hSCARB2-tg mice were inoculated with EV-A71 B5 (19 strains) intraperitoneally at 5 x 10^5 $TCID_{50}$. The B5 strains were islolated from the patients with diffrent clinical grade. Of them, 10, 8 and 1 strains were from grade 1, grade 2 and grade 4 of B5-infected patients, respectively. A: Paralysis rates at 14 days post-infection. B: Mortality rates at 14 days post-infection. P-values were calculated with the Mann-Whitney U test. EV: enterovirus, hSCARB2-tg mice: mice that carry the human scavenger receptor class B2 transgene.

Supplementary Figure S1

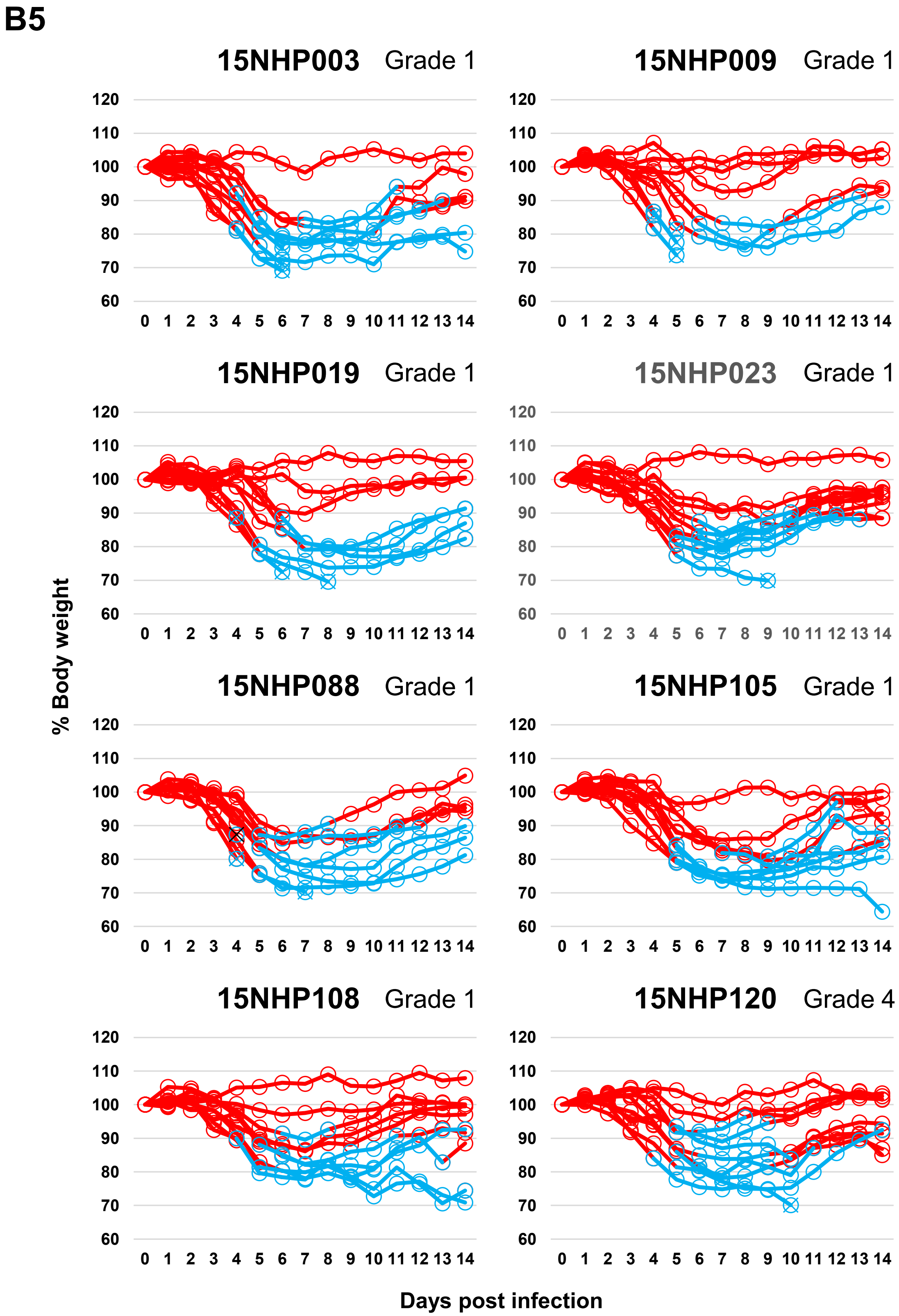

B5

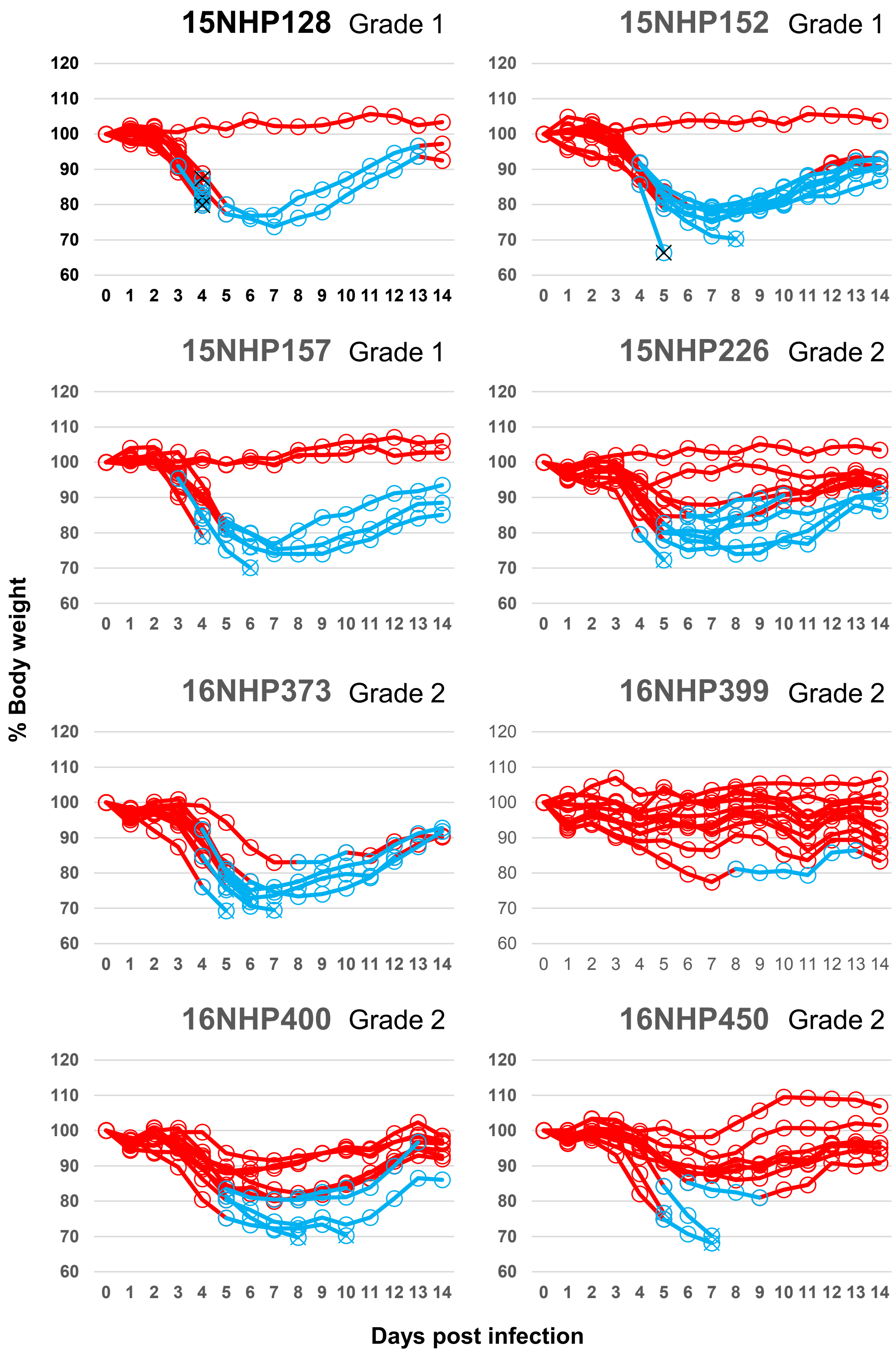
15NHP128 Grade 1
15NHP152 Grade 1
15NHP157 Grade 1
15NHP226 Grade 2
16NHP373 Grade 2
16NHP399 Grade 2
16NHP400 Grade 2
16NHP450 Grade 2
% Body weight
Days post infection
60
70
80
90
100
110
120
0 1 2 3 4 5 6 7 8 9 10 11 12 13 14

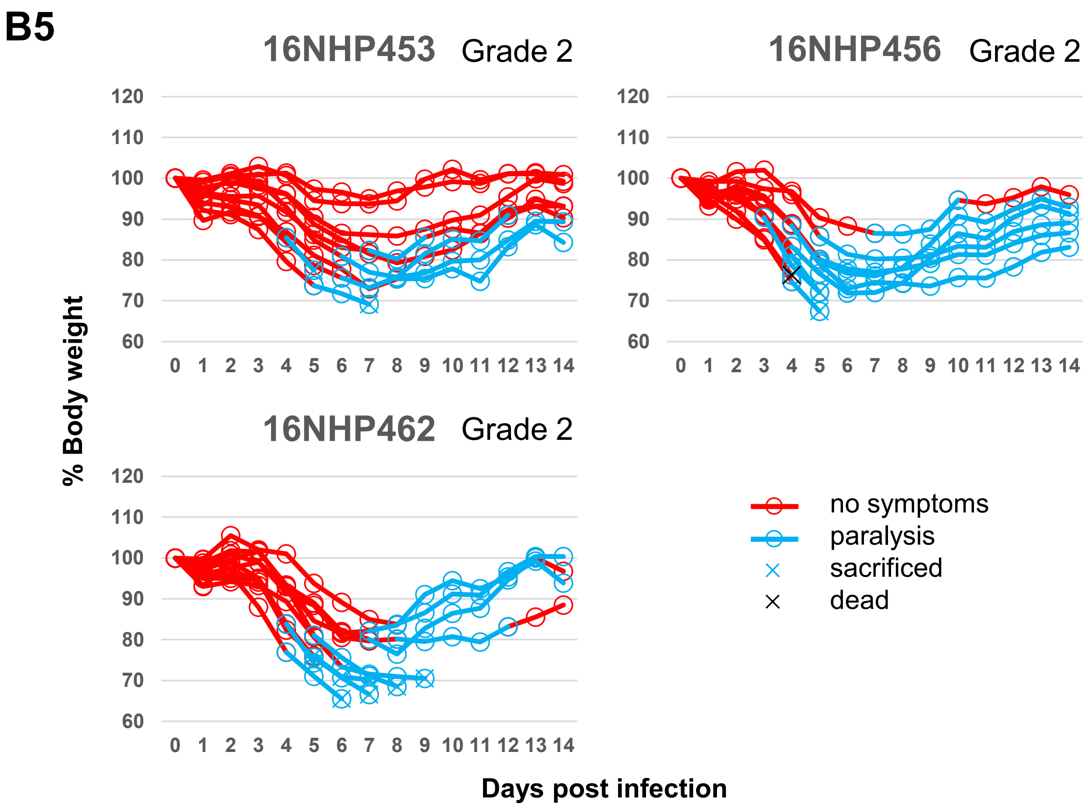
B5
16NHP453 Grade 2
16NHP456 Grade 2
16NHP462 Grade 2
% Body weight
120
110
100
90
80
70
60
0 1 2 3 4 5 6 7 8 9 10 11 12 13 14
Days post infection
no symptoms
paralysis
sacrificed
dead

C4

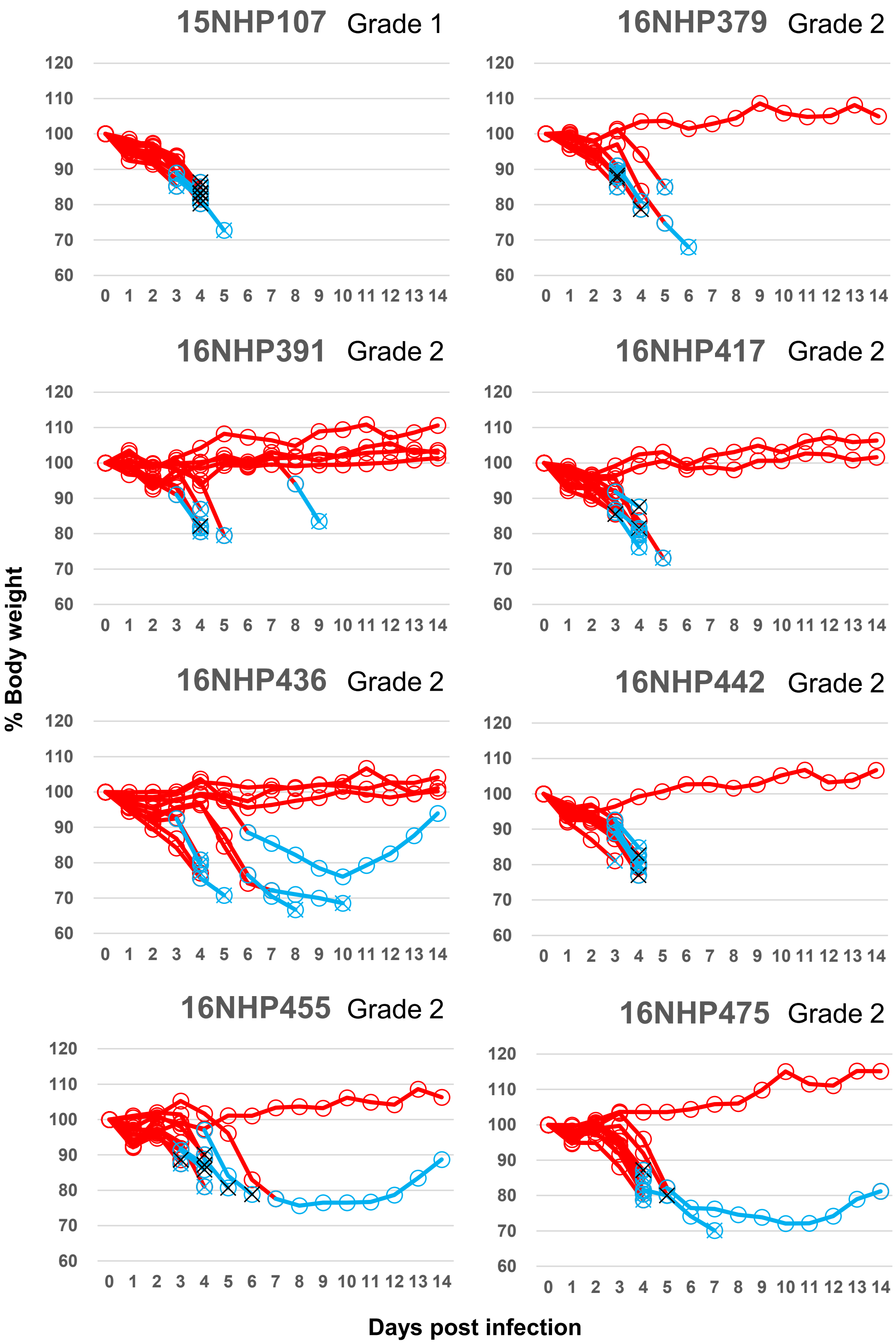

15NHP107 Grade 1
16NHP379 Grade 2
16NHP391 Grade 2
16NHP417 Grade 2
16NHP436 Grade 2
16NHP442 Grade 2
16NHP455 Grade 2
16NHP475 Grade 2
120
110
100
90
80
70
60
0 1 2 3 4 5 6 7 8 9 10 11 12 13 14
% Body weight
Days post infection

Supplementary Figure S2

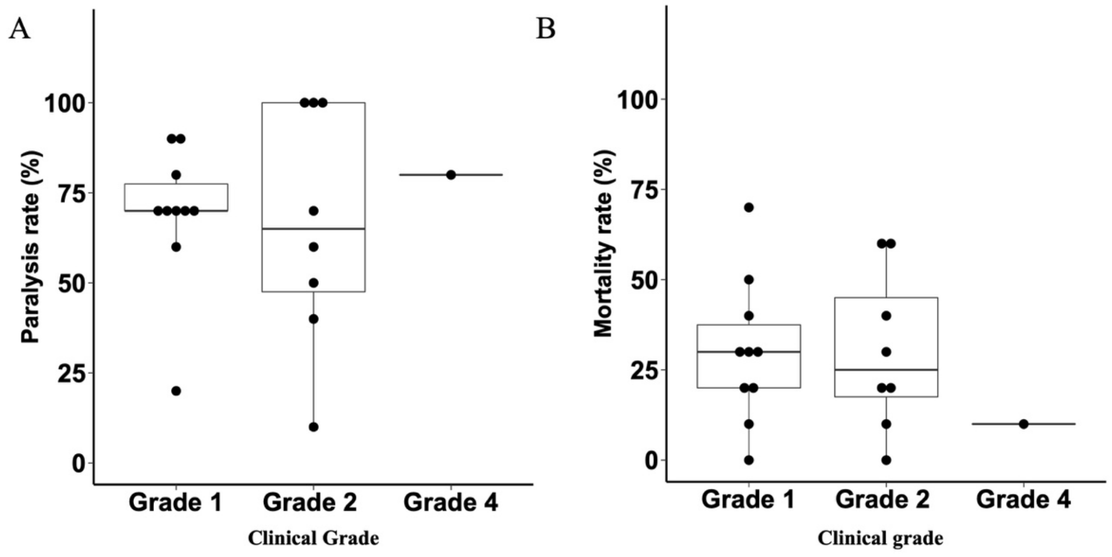

Supplementary Table S1. Summary of the next generation sequencing analysis of the EV-A71 strains

virus	Subgenogroups	mapped_reads	average_depth	Abundance of VP1-145 mutation (%)
15NHP002	B5	307823	5839.17	0.78*
15NHP003	B5	696335	7657.62	0.00
15NHP009	B5	635531	7643.31	0.00
15NHP019	B5	623879	7605.05	0.00
15NHP023	B5	390067	6849.36	0.00
15NHP088	B5	829751	7725.44	0.00
15NHP105	B5	239500	4650.57	0.00
15NHP108	B5	182415	3550.84	0.00
15NHP120	B5	260063	4972.82	0.00
15NHP128	B5	275994	5304.13	0.00
15NHP152	B5	169800	3266.71	0.00
15NHP157	B5	437939	7134.11	0.00
15NHP226	B5	173209	3358.33	0.00
16NHP373	B5	226537	4352.49	0.00
16NHP399	B5	1037217	7777.11	0.00
16NHP400	B5	501407	7377.05	0.00
16NHP401	B5	1011903	7786.68	1.65*
16NHP415	B5	1206752	7807.39	1.55*
16NHP446	B5	955315	7785.03	1.71*
16NHP450	B5	1003009	7789.88	0.00
16NHP453	B5	1375485	7838.46	0.00
16NHP456	B5	1383746	7832.95	0.00
16NHP459	B5	1444424	7839.17	1.39*

16NHP462	B5	833024	7782.74	0.00
15NHP107	C4	291161	5695.18	0.00
16NHP379	C4	191191	3784.55	0.00
16NHP391	C4	223860	4421.73	0.00
16NHP417	C4	291526	5736.53	0.00
16NHP436	C4	483159	7341.13	0.00
16NHP442	C4	310591	6070.31	0.00
16NHP455	C4	195836	3868.44	0.00
16NHP475	C4	231924	4564.31	0.00

*amino acid mutation to Glycine was detected.
